# On the lag phase in amyloid fibril formation

**DOI:** 10.1039/c4cp05563b

**Published:** 2015-02-26

**Authors:** Paolo Arosio, Tuomas P. J. Knowles, Sara Linse

**Affiliations:** a Chemistry Department , University of Cambridge , Lensfield road , Cambridge , UK; b Department of Biochemistry and Structural Biology , Chemical Centre , Lund University , P. O. Box 124 , SE221 00 Lund , Sweden . Email: sara.linse@biochemistry.lu.se

## Abstract

The formation of nanoscale amyloid fibrils from normally soluble peptides and proteins is a common form of self-assembly phenomenon that has fundamental connections with biological functions and human diseases. The kinetics of this process has been widely studied and exhibits on a macroscopic level three characteristic stages: a lag phase, a growth phase and a final plateau regime. The question of which molecular events take place during each one of these phases has been a central element in the quest for a mechanism of amyloid formation. In this review, we discuss the nature and molecular origin of the lag-phase in amyloid formation by making use of tools and concepts from physical chemistry, in particular from chemical reaction kinetics. We discuss how, in macroscopic samples, it has become apparent that the lag-phase is not a waiting time for nuclei to form. Rather, multiple parallel processes exist and typically millions of primary nuclei form during the lag phase from monomers in solution. Thus, the lag-time represents a time that is required for the nuclei that are formed early on in the reaction to grow and proliferate in order to reach an aggregate concentration that is readily detected in bulk assays. In many cases, this proliferation takes place through secondary nucleation, where fibrils may present a catalytic surface for the formation of new aggregates. Fibrils may also break (fragmentation) and thereby provide new ends for elongation. Thus, at least two – primary nucleation and elongation – and in many systems at least four – primary nucleation, elongation, secondary nucleation and fragmentation – microscopic processes occur during the lag phase. Moreover, these same processes occur during all three phases of the macroscopic aggregation process, albeit at different rates as governed by rate constants and by the concentration of reacting species at each point in time.

## Abstract 2

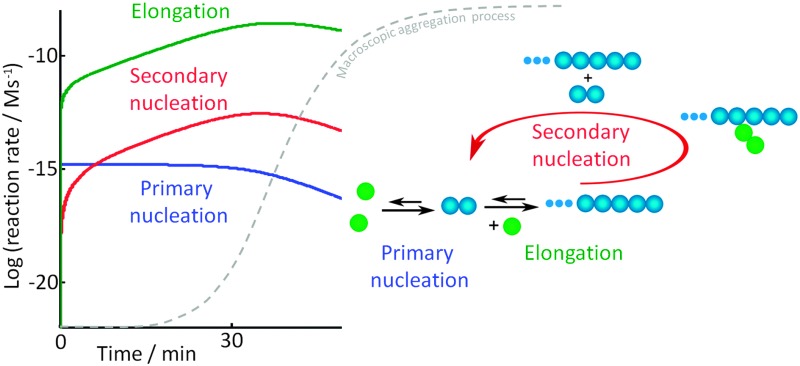
Rates of microscopic processes taking place during the lag phase of amyloid fibril formation for a reaction starting from an initially monomeric 4 μm solution of Aβ42.

## 1. Introduction

**Fig. 1:**
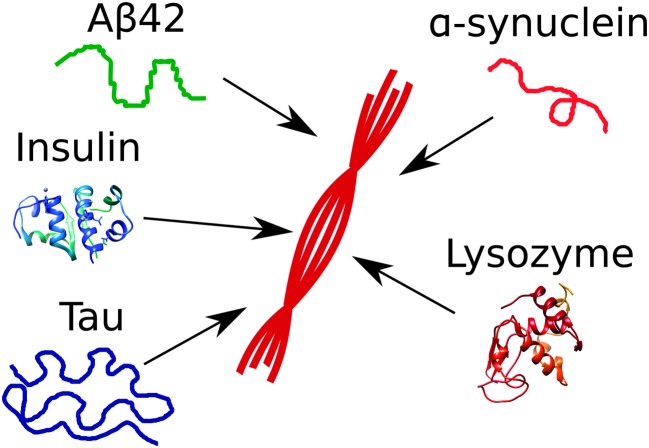
Sketches of amyloid forming proteins associated to several human diseases.

Amyloid fibrils are observed in connection with more than 30 human diseases that are increasingly prevalent yet currently incurable in the vast majority of cases. Well-known examples are Amyloid Lateral Sclerosis (ALS), Alzheimer, Parkinson, Huntington's diseases and diabetes.^1–4^ Amyloid has also emerged as a functional state in bacteria and fungi,^5^ as well as in humans.^6–8^ Such functionality is thought to originate from the fact that this stage offers a route towards the very tight packing of proteins, which provides a stable storage environment, favorable mechanical properties and in some cases an active catalytic surface for biosynthetic pathways.^9^ From an ultra-structural point of view, amyloid fibrils are highly ordered and elongated aggregates characterized at the molecular level by the presence of an array of β-strands oriented perpendicularly to the long axis of the fiber.^10–12^ Despite great variation in sequence length and native, or natively unfolded, structure of the precursor proteins, once assembled into amyloid fibrils, they possess highly similar generic structures but with some variation in local packing leading to morphological differences in, for example, twist and internal lateral packing of β sheets (Fig. 1).

Thus, the amyloid structure is likely to represent an alternative stable structure for peptides and proteins accessible to most if not all peptides and proteins.^13,14^ An individual amyloid fibril is often formed from a protein with a given core sequence and the structure is highly repetitive with the same inter-protein contacts stabilizing the strands throughout the length of the fibril.^10,11^ Amyloid deposits found *in vivo* in the context of disease may, however, contain more than one protein, or protein variant, a fact that may be due to association of fibrils or association of other proteins to the fibrils.^15^ Amyloid deposits can also be rich in lipids.^16^ For many amyloid-forming proteins, it has been found that monomers and full-length fibrils induce limited or no toxicity to neurons or other cells.^17^ In contrast, cell death can be induced by smaller aggregates of intermediate sizes forming during the amyloid growth process.^18,19^ The size-distribution and structures of these toxic assemblies are not established, although some hints are emerging.^20–23^ It has also been proposed that the aggregation process *per se* is toxic.^24^ In all cases, cellular toxicity is associated with an ongoing assembly process in which free monomers are converted to the amyloid state. From a biological and biomedical point of view, it is therefore of key importance to understand the amyloid formation process in terms of the underlying molecular events that define its mechanism.

Such a mechanistic understanding implies a dissection of the overall aggregation process into its underlying composite reactions, the microscopic steps. Moreover, important insights may be gained by defining the rate constants governing each microscopic step and determining the manner in which they depend on protein sequence as well as on solution conditions such as salt, pH, temperature, other proteins, membranes, and species that inhibit protein aggregation.

One relevant feature of the aggregation process of amyloids is the lag-phase that is commonly observed during the kinetics of fibril formation. The molecular origin of this lag-phase has historically attracted large attention because of the key mechanistic information that is potentially contained in this macroscopic observable. In this review, after presenting the currently available experimental evidences, we discuss the microscopic mechanisms underlying the origin of the lag-phase, providing a mechanistic framework to dissect information on the microscopic aggregation steps from this measurable macroscopic phase. We elucidate most of the concepts discussed in this work using as an example the Alzheimer's disease-associated peptide Aβ42, for which a detailed quantitative and mechanistic picture of the aggregation process is now available. However, the concept of lag-phase discussed here is general, and applies to other amyloidogenic peptides and proteins.

## 2. Monomers, aggregates, fibrils, oligomers and nuclei

**Fig. 2:**
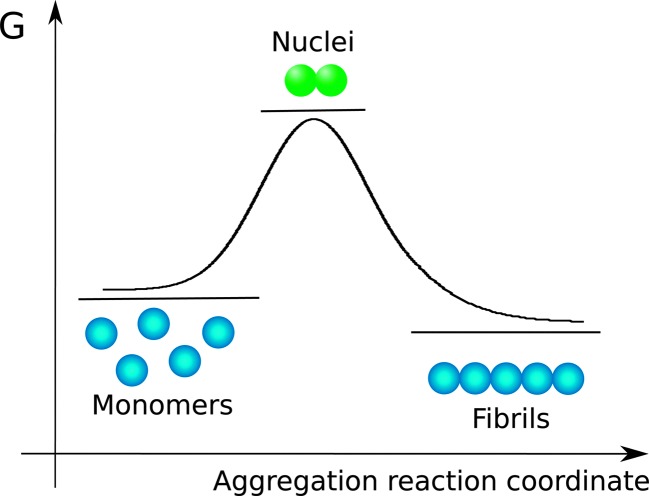
Free energy diagram of amyloid fibril formation. The nucleus is the state with the highest free energy. Fibrils and monomers may have similar free energy, and the total concentration of monomer governs which state dominates at equilibrium.

The species observed to form as a consequence of protein aggregation processes are commonly highly heterogeneous. Indeed, the overall reaction results in the conversion of monomeric soluble peptides or proteins into an array of aggregate structures; in large part due to this significant level of heterogeneity, a standard terminology to describe specific aggregate species has not yet emerged; in the following, a monomer is defined as a single protein or peptide chain. Monomers may exist free in solution or as complexes with other monomers in aggregates. With aggregate we denote any assembly containing more than one monomer, *i.e.* dimers, trimers and higher order assemblies. Amyloid fibrils are linear aggregates with a repetitive cross-beta structure which may consist of tens of thousands of monomers. These structures are able to elongate rapidly from their ends by association of peptides or proteins from the solution phase. By contrast, many smaller aggregates, commonly termed oligomers, elongate significantly more slowly than amyloid fibrils. The definitions for oligomers vary in the literature. This term is often used to refer to aggregates up to a certain size, *e.g.* 2–20-mers. Here, the upper limit is not always given, and different limits may be of practical value for different systems. The significantly lower rates of growth process for oligomers compared to amyloid fibrils suggest that there are significant structural differences between amyloid fibrils and oligomers. Indeed, among the several possible definitions, oligomers can be described as small aggregates exhibiting different structure and lower growth rate with respect to fibrils. It has been challenging, however, to define the smallest size of an aggregate that possesses the characteristics of an amyloid fibril rather than of an oligomer, since rapid elongation makes the shortest fibrils extremely short-lived and difficult to observe. In some reports, oligomers are defined according to preparation method, or in terms of which substances are added to block them from further growth. In this report we will use the term oligomer to define the small aggregates that are generated by the first few elongation events of nuclei without any reference to their structure. Nuclei are the smallest aggregates in the process that are stable enough that further growth by monomer addition is faster than dissociation into monomers. In energetic terms, nuclei are thus the species with highest Gibbs free energy (Fig. 2) along the aggregation pathway. We note that the schematic shown in Fig. 2 is only a simplified version of the complex multi-dimensional free energy diagram associated to the formation of amyloid fibrils. In addition, nucleation reactions follow complicated pathways that populate a cascade of intermolecular metastable species.^25^

It is worth pointing out that in the figures of this work, monomeric units are illustrated as spheres without inference to their structures, because in the frame of the mechanistic models discussed in this paper energetic diagrams and reaction schemes are described in a coarse-grained approach based on the chemical potentials and the microscopic rate constants associated to co-existing species. The chemical potentials and the rate constants summarize the global contribution of all the detailed molecular features of the monomeric units, which are ultimately responsible for the aggregation propensity.

## 3. Kinetics of amyloid fibril formation

**Fig. 3:**
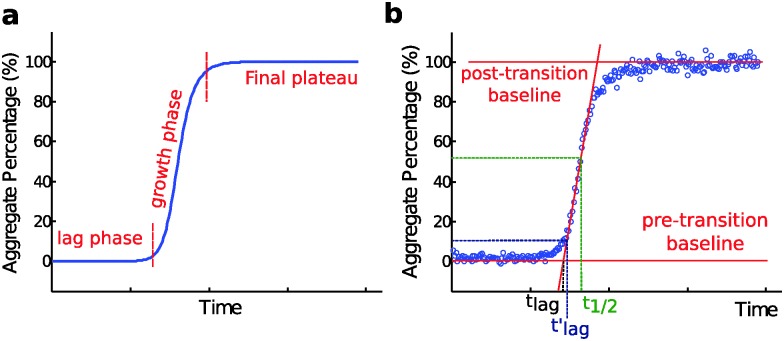
(a) A characteristic macroscopic aggregation curve for amyloid fibril formation is displayed in terms of aggregate concentration (in monomer equivalents, % of total monomer) *versus* time. The curve is typically divided into a lag phase, a growth phase and a final plateau; (b) definition of *t*_1/2_, and two alternative definitions of the lag time. Here *t*_lag_ is obtained by extrapolating the maximum derivative down to the intercept with the pre-transition base-line; while *t*_lag_′ and *t*_1/2_ are defined as the point in time where the signal relative to the pre-transition base line has reached 10% and 50% of the amplitude of the transition, respectively.

Nucleated self-assembly reactions, including amyloid fibril formation, typically display sigmoidal growth kinetics.^26–28^ A steep transition zone is both preceded and followed by relatively flat regions (Fig. 3a). The region before the transition zone is referred to as the lag phase. The steep transition zone is often called the growth phase or elongation phase as the overall conversion rate of peptides or proteins into their amyloid forms is greatest in this part of the reaction. The final flat region is known as the plateau phase and represents a steady state where the monomer concentration has reached its equilibrium value. The onset and end of the transition can be more or less sharp depending on the dominant underlying mechanism,^29^ as discussed further below. Importantly, however, none of these three phases can be ascribed to a single molecular event or microscopic process.^26–29^ Rather, all microscopic processes are ongoing during all phases of the process, although their net flux varies as governed by rate constants and the activities of reacting species at each particular point in time. In dilute samples, activities are approximated by concentrations.

## 4. Definition of the lag time

The duration of the lag phase is called the lag time, *t*_lag_. However, there are multiple ways to define the cross-over between the lag phase and the growth phase and thus investigators use different methods to extract *t*_lag_ from experimental data. One common definition of *t*_lag_ is the point in time where the signal relative to the pre-transition base line has reached a chosen fraction (*e.g.* 10% or 50%) of the amplitude of the transition (Fig. 3b). If 50% is used the time point is often called the half time, *t*_1/2_ or *t*_0.5_, rather than *t*_lag_. Another common method involves finding the maximum derivative and extrapolating down to the intercept with the pre-transition base-line (Fig. 3b). We will see below that for many classes of growth reactions, in particular those dominated by secondary nucleation, the threshold and the extrapolation approaches result in very similar values for the lag time of the reaction, and in particular both definitions lead to a lag time that has an identical scaling behavior in response to changes in the concentration of the precursor peptide or protein in solution at the beginning of the reaction.

In some studies, an empirical sigmoidal function (logistic function) is fitted to the data*y* = *y*_0_ + *A*/(1 + exp(–*k*(*t* – *t*_0.5_)))where *y*_0_ is the pre-transition base line, *A* the amplitude of the transition, *t*_0.5_ its midpoint and *k* is an apparent growth rate. By this method the lag time is often defined as *t*_lag_ = *t*_0.5_ – 1/2*k* which is equivalent to the extrapolation from the maximal growth rate, Fig. 3b. The logistic function is not routed in a specific molecular level process underlying amyloid formation and a number of other functions can also be used to fit sigmoidal data. It has become apparent therefore, that a strong test of a proposed reaction mechanism by comparing rate laws with experimental data is only possible when data acquired over a large concentration range are fitted in a global manner.^29^

## 5. Methods to determine the lag time

A number of experimental methods can be used to follow in a fully quantitative manner the kinetics of amyloid formation and thus allow the lag time to be determined. These methods monitor the decrease in the concentration of the monomeric species or the appearance of aggregates, and can be divided into methods that operate *in situ*, *i.e.* during the reaction, and methods that operate *ex situ*, *i.e.* require post-handling of aliquots taken from the aggregating mixture at well-defined times. Many of these methods are susceptible to experimental artifacts, and in order to obtain fully reliable data, it is important to verify the reproducibility of the data for multiple replicates of the reaction initiated from the same precursor solution and to check with at least one independent method that the observed changes in a signal are a faithful representation of the aggregation time course. In addition, these methods can be complemented by imaging techniques, such as atomic force microscopy (AFM)^30,31^ or cryo-transmission electron microscopy (cryo-TEM),^32^ which can provide relevant qualitative and semi-quantitative information on the fibril formation process.

### 5.1 Circular dichroism spectroscopy

**Fig. 4:**
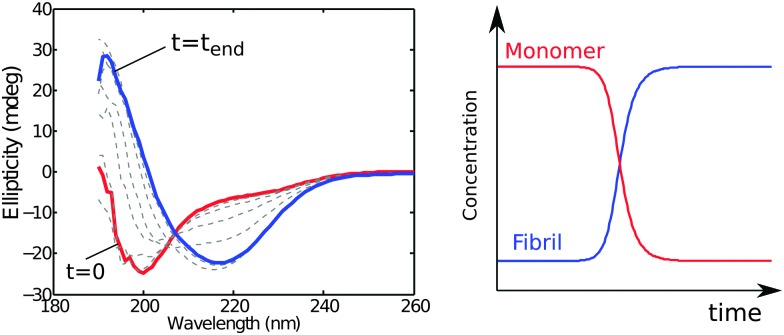
CD spectra acquired during an ongoing reaction (left). The first spectrum (red) shows the unfolded monomer and the last spectrum (blue) the β-sheet fibril. The monomer and fibril concentration as a function of time (right) can be extracted by fitting superpositions of the start and end spectra to the experimental data acquired at different time points.

The monomer concentration may be monitored *in situ* using circular dichroism (CD) spectroscopy relying on the different characteristic spectra corresponding to different secondary structures or random coil conformations of proteins. Thus, monomer conversion is monitored by following the change in the random coil signal from unstructured monomers (Fig. 4) or other characteristic signals if monomer has some degree of folding. The aggregate concentration may also be determined *in situ* using CD spectroscopy by exploiting the fact that the latter species are β-sheet rich (Fig. 4).^33^ An advantage of this method is that monomer and fibril concentration can be quantified simultaneously in the same sample at each time point by fitting superimpositions of the start and end spectra.

### 5.2 NMR spectroscopy

**Fig. 5:**
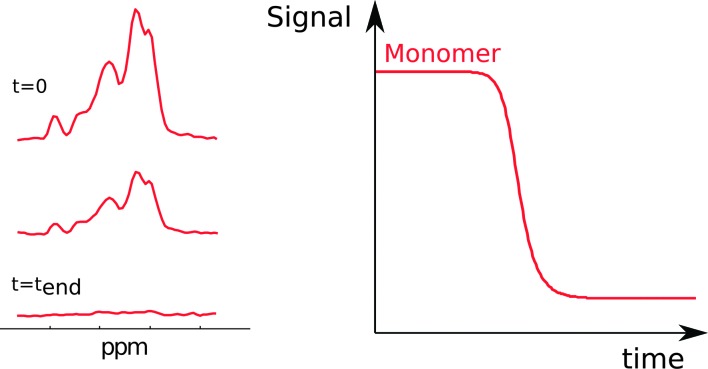
NMR spectra acquired during an ongoing reaction (left). The monomer concentration as a function of time (right) is extracted from the peak intensities.

Another method to monitor *in situ* the concentration of monomer is nuclear magnetic resonance (NMR) spectroscopy (Fig. 5). This method relies on the fact that at least some chemical shifts of protons, nitrogens-15 or carbons-13 in the protein have unique values in monomers. The sharp signals from fast tumbling entities like small peptides and proteins indicate that monomers are readily detected, whereas in aggregates only mobile regions can be monitored. The monomer concentration can be deduced from the intensity of monomer signals.^34,35^ Due to the low value of the gyromagnetic ratio for nuclei, reliable monitoring of the aggregation process by NMR requires relatively high peptide or protein concentrations, commonly in the micromolar range.

### 5.3 Infrared spectroscopy

The aggregate concentration can be monitored *in situ* using Fourier transform infrared (FTIR) spectroscopy relying on the characteristic vibration frequency of hydrogen bonded back-bone amides in extended β-sheet structure.^36^ FTIR spectra are conventionally represented in terms of absorbance intensity as a function of wave numbers, defined as the reciprocal of the wave lengths, with units of cm^–1^. For proteins, the major band of interest is the amide I, which absorbs in the region 1600–1700 cm^–1^. Different secondary structures are characterized by different spectra. In particular, FTIR spectra of β-sheet structures exhibit a maximum in the region 1615–1643 cm^–1^. As a consequence, fibril formation can be followed by monitoring the increase in the intensity in this characteristic range during time, but this technique requires high protein concentration.

### 5.4 Fluorescence spectroscopy

**Fig. 6:**
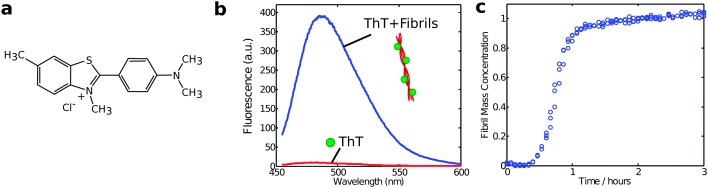
(a) Structure of the ThT dye and (b) change in the emission fluorescence spectrum upon binding to amyloid fibrils. Typical excitation wavelength is 440 nm; (c) the fibril formation process is monitored by recording the relative changes in the fluorescence intensity during time with respect to the situation at time zero.

Another method to monitor *in situ* the aggregate concentration is intrinsic fluorescence spectroscopy relying on specific spectral changes upon amyloid formation,^37^ or indirectly by the fluorescence from the reporter dye thioflavin T, which undergoes a dramatic enhancement of quantum yield when bound to fibrils (Fig. 6).^38,39^ ThT fluorescence was historically one of the first assays to be used to probe the kinetics of protein aggregation and remains one of the most widely used experimental approaches for this purpose. Yet, despite its longstanding and widespread use, it remains challenging to obtain fully quantitative data using ThT fluorescence assays. This difficulty is in large part due to the fact that the molecular details of the binding of ThT to amyloid fibrils are not fully known and that the resulting fluorescence intensity is susceptible to perturbations from the presence of impurities or amorphous protein aggregates in the system. Furthermore, the fluorescence signal is linearly dependent on the concentration of aggregates only in a relatively small range of ThT and protein concentrations, which need to be optimized in each study.^17^

### 5.5 Scattering methods

Scattering methods, such as static and dynamic light scattering and small angle X-ray scattering may be used *in situ*.^20,40–48^ These methods rely on the fact that the scattering intensity is highly dependent on the particle size. As a consequence, the scattering properties of aggregates are significantly larger compared to monomers, and the formation of fibrils can be detected by following the increase in the scattered intensity during time. In addition, the dependence of the scattered intensity on the scattering angles contains key information about the shape of the objects. However, in these approaches it may be challenging to resolve information on individual species within heterogeneous complex mixtures from the recorded average signal, since this procedure requires a deconvolution process that is a well-known ill-posed problem highly sensitive to experimental noise. Moreover, average quantities of the aggregate distribution could be significantly biased towards larger species because of the high dependence of the scattered intensity on the particle radius, as described by the Rayleigh formalism. As a consequence, for heterogeneous mixtures it is challenging to apply these methods in a fully quantitative manner.

### 5.6 *Ex situ* methods

In an *ex situ* approach, aliquots of a fixed volume are collected from an aggregating reaction mixture at different times during the aggregation process and separated for analysis. The advantage of such approach relative to *in situ* method is the possibility to use techniques that would perturb the aggregation reaction, since the aliquots have been physically separated from the reaction mixture and their treatment thus does not affect the ongoing reaction. In particular, fractionation into monomer, fibrils and possibly also smaller aggregates at different time points along the reaction can be achieved using centrifugation, filtration, chromatography, electrophoresis, or other analytical techniques, and the quantification of individual species can exploit for example absorbance, UV-Vis, IR fluorescence, radioactivity, mass spectrometry with internal standard, or antibody interactions.^17,43,49^

### 5.7 Amyloid chain amplification methods

A particularly sensitive *ex situ* assay for detecting the presence of amyloid fibrils is that of the amyloid chain amplification approach.^32,50^ In this method, aliquots are taken from a reaction mixture at different times, and the fibrils contained in these aliquots are isolated by means of filtration (Fig. 8). The concentration of fibrils trapped in this manner is quantified by adding fresh monomer solution to the retentate and comparing the resulting aggregation kinetics, monitored through ThT fluorescence, with a calibration curve obtained using controlled amounts of fibril seeds to initiate the aggregation of a solution of monomer at the same concentration. Due to the exponential amplification step inherent in this approach, sensitivities of at least two orders of magnitude higher than with ThT fluorescence alone have been demonstrated. This method has revealed that the fibril concentration grows with a close to exponential rate during the lag phase, *i.e. C*_fibrils_ *= A*(cos*h*(*kt*) – 1), where *t* is time, *A* is a parameter related to the nucleation of new fibrils and *k* is an effective polymerization rate constant which reflects the multiplication and growth rates of fibrils.^32^

## 6. The major species in solution are monomers or fibrils at all times

**Fig. 7:**
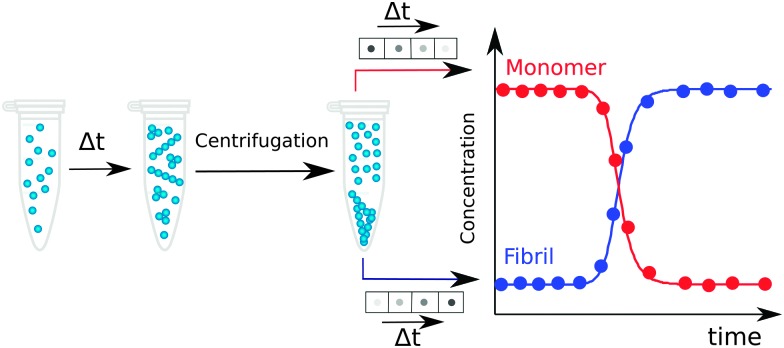
An example of post-reaction analysis of monomer and aggregate concentration *ex situ*. Samples are withdrawn from an ongoing reaction, separated into monomers and fibrils by centrifugation, and quantified using immunoblots, UV absorbance or ELISA assay.

Work over the past decade in different laboratories using many of the methods listed above has revealed a picture of the aggregation process in which monomers or fibrils are the dominating species during the entire time course of the aggregation process. Monomers are the prevalent species during the lag phase and fibrils dominate at the final plateau, while during the growth phase their concentrations are similar. If the monomer and fibril concentration are both measured and plotted in monomer equivalents, the curves corresponding to the time evolution of the decrease in the monomer concentration and the increase in the fibril concentration follow specular sigmoidal shape, and they cross at a time point very close to the half-time, where the concentrations of monomer and fibril are close to 50% each (Fig. 4b and 7). The concentrations of any intermediates, small aggregates or oligomers, are low at all times. As a consequence, the concentrations of intermediate species are challenging to measure, although they can still be determined for instance by *ex situ* measurements using radio-isotope labelling, separation of the reaction mixture by size exclusion chromatography and post-analysis by radio-counting of oligomeric *versus* monomeric fractions. In the case of Aβ42, such approach revealed that the total concentration over all oligomers (3–20mers) is always less than 1.5% of the initial monomer load, with the maximum appearing at the point in time where the monomer and aggregate curves cross.^17^ Dimer quantification is impossible by this method as dimers elute in the tail of the monomer peak.

**Fig. 8:**
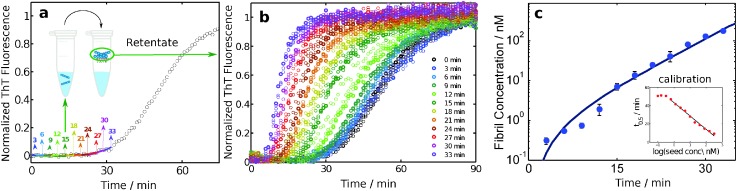
An amyloid chain amplification method. Samples are withdrawn from an ongoing reaction and separated on 200 nm filters (a). The retentates are added to fresh monomer and the aggregation kinetics, monitored through ThT fluorescence (b), are compared to reactions seeded with controlled amounts of fibrils at the same monomer concentration (c).^32^

## 7. Molecular events during the lag time

**Fig. 9:**
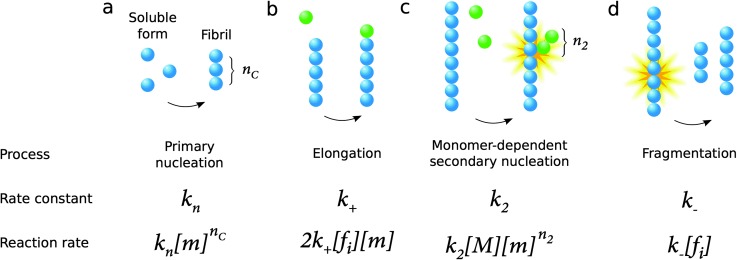
Microscopic processes underlying amyloid formation and associated rate constants and reaction rates: primary nucleation from monomers in solution (a), elongation (growth) by monomers addition to existing aggregates (b), surface catalyzed secondary nucleation from monomers on fibril surface (c) and fragmentation (d). In the expressions of the reaction rates in the last row, [*m*] refers to the free monomer concentration, [*M*] to the total fibril mass concentration and [*f*_i_] to the fibril number concentration, while the rate constants are defined in the middle row.

The process of amyloid formation, when initiated from a solution of the monomeric precursor protein, requires fundamentally at least two microscopic steps: primary nucleation from monomers in solution and elongation of fibrils, a process by which monomers add to the ends of existing aggregates leading to their growth (Fig. 9). Since the growth of fibrils occurs from their ends, any process that is susceptible to modify the number of fibril ends in the system has a significant impact on the overall aggregation kinetics. In this context, a microscopic step that has been found in several systems under agitation conditions is fragmentation (Fig. 9). Moreover, surface catalyzed secondary nucleation from monomers on fibril surface may also play a key role for several systems under quiescent conditions (Fig. 9).

As mentioned above, all microscopic processes underlying the overall reaction are active during all phases of the reaction.^26–29^ Thus, none of the three phases seen in the overall aggregation curve – lag phase, growth phase and final plateau (Fig. 3) – can be ascribed to a single microscopic process as the laws of mass action do not allow a discontinuity in the reaction rates of any microscopic processes. A particularly interesting case is that of primary nucleation; in a system which contains initially a solution of purely monomeric peptides or proteins, the concentration of aggregates is identically 0 at very early times, and thus the fibril-dependent processes (elongation, fragmentation and secondary nucleation) are completely suppressed, and primary nucleation is the only molecular level process contributing to amyloid formation that is active under these conditions. Crucially, however, the values of the rate constants that are obtained from a quantitative analysis of amyloid growth kinetics reveal that under bulk conditions, initial nuclei are formed through primary nucleation within milliseconds, and thus the time span where only primary nucleation takes place extends typically *ca.* 10^–7^% into the lag time; after this point also elongation can occur, and soon afterwards also secondary nucleation events. Additional processes, including fragmentation, may occur not only if samples are agitated, but also in quiescent samples depending on the stability of the fibrils, which may vary with solution conditions. In the case of Aβ42 and Aβ40 under quiescent conditions, three processes occur with significant rate: primary nucleation elongation, and secondary nucleation.

### 7.1 Millions of primary nuclei may form during the lag phase

**Fig. 10:**
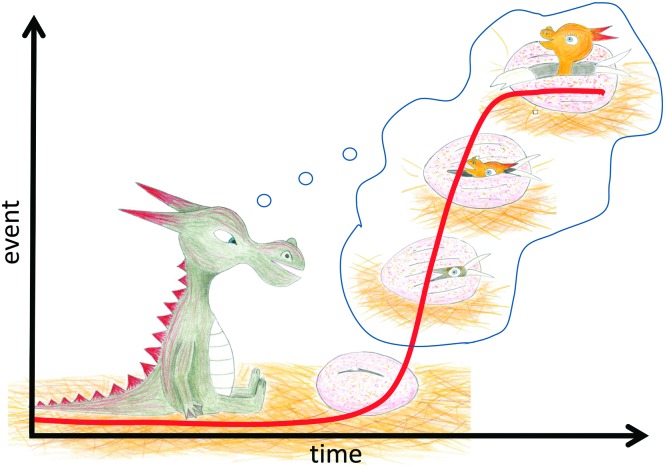
We are used to think in small numbers in a linear fashion. This vision may induce the misleading interpretation of the lag phase as a waiting time for fibrils to appear from a small number of oligomers. However, typical samples used in amyloid studies contain billions of monomers or more, and millions of primary nuclei may form during the lag phase. Adapted from ref. 51.

The lag time for fibril formation is sometimes called the nucleation period, a fact which could lead to the inference that this time represents a waiting time for the first nuclei to appear. This picture is sometimes seen in the literature, and may have emerged because in every-day life we are used to think in small numbers (Fig. 10).

As discussed above, however, this type of collective waiting behavior is not in agreement with the laws of mass action and may be possible only for single-molecule reactions. We discuss here the lag phase in macroscopic samples studied by bulk methods as listed above. Typical concentrations used in these studies are 1 nM–100 μM and the sample volumes are typically 1–500 μL. Those samples thus contain 6 × 10^8^–3 × 10^16^ monomers. In such large samples, the reaction is governed by rate constants and activities, which could be well approximated by concentrations in the low concentration regime (Fig. 9). The large number of molecules present in such systems suggests that statistical fluctuations should not manifest in any key observables and that the reaction time course will be the same each time an identical sample is studied.^49,52^ As discussed above, the first nuclei appear during the very early part of the lag phase. As an example, we consider a 100 μL solution of 4 μM Aβ42, a peptide implicated in Alzheimer's disease, which at *t* = 0 is purely monomeric. The lag time has been measured to be 33 minutes = 2000 seconds.^17,32^ Using the rate constant for primary nucleation as derived from kinetics analysis of a large experimental data set,^17,53^ *k*_*n*_ = 3 × 10^–4^ M^–1^ s^–1^, we can calculate the number of primary nuclei formed during the first second (*i.e.* the first 0.05% of the lag phase) as 1 s × 3 × 10^–4^ M^–1^ s^–1^ × (4 × 10^–6^ M)^2^ × 10^–4^ l × 6 × 10^23^ mol^–1^ = 3 × 10^5^. The inverse of this rate represents a time scale for the average time for the appearance of the first nucleus as 3 μs, which is 10^–7^% of the lag time. Since the monomer concentration stays close to 4 μM during the entire lag phase, the generation of primary nuclei occurs with a rate that is approximately constant until the end of the lag phase when monomer depletion due to the growth of aggregates becomes significant. The number of primary nuclei generated during the lag phase of our example can thus be calculated as 2000 × 3 × 10^5^ = 6 × 10^8^. In other words, 600 million primary nuclei have been formed before the end of the lag phase is reached.

### 7.2 Secondary nuclei outnumber primary nuclei

**Fig. 11:**
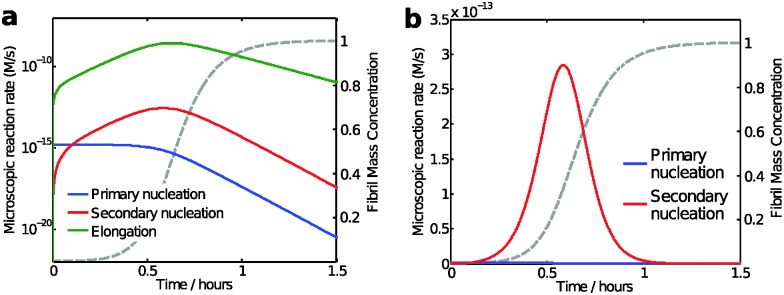
Model predictions of the aggregation reaction of a 4 μM solution of Aβ42 using the rate constants as determined from kinetic analysis of a large body of data;^17,53^ (a and b) microscopic reaction rates: the maximum elongation (green line) and secondary nucleation rate (red line) occurs close to the half-time, while primary nucleation rate (blue line) is constant during the lag phase and decreases as monomers concentration is reduced. The reaction rates are shown with logarithmic *y*-axis in (a) and with linear *y*-axis in (b). The macroscopic aggregation curve is shown as a dashed grey line with linear *y*-axis in both panels.

While the calculation discussed above for the rate of formation of primary nuclei shows that a very large number of primary nuclei are generated during the lag phase, they may be outnumbered by nuclei generated by secondary nucleation reactions catalysed by the fibril surface (Fig. 11). Such fibril-catalysed nucleation has been observed in the cases of Aβ42^17^ and Aβ40,^53^ as well as α-synuclein involved in Parkinson's disease.^54^ The elongation rate, 2*k*_+_[*f*_i_][*m*], governing the growth of nuclei towards long amyloid fibrils is typically much higher than the rate for primary nucleation, *k*_*n*_[*m*]^*n*_C_^ (Fig. 11a). Therefore, the first fibrils appear almost immediately after the first nucleation event. These fibrils provide a catalytic surface for nucleation and because of the high rate constant, *k*_2_ = 10^4^ M^–2^ s^–1^, the number of nuclei generated by secondary nucleation will dominate over primary nuclei as soon as the fibril concentration exceeds a threshold concentration, [M*]. This concentration can be calculated as
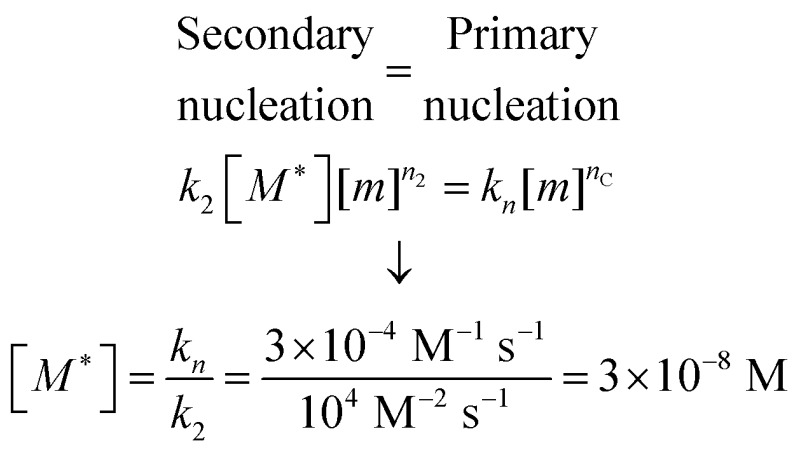
being *n*_C_ = *n*_2_ = 2 in the case of Aβ42.^17^ Thus, when the aggregate concentration reaches 30 nM, the secondary nucleation rate is higher than the primary nucleation rate, as shown by the cross of the red and blue curves in Fig. 11a. When the nucleation rates are shown on a linear scale (Fig. 11b) the cross is barely visible and the much higher rate of secondary compared to primary nucleation during most of the reaction time course is evident. Since both monomers and fibrils are reactants for the secondary nucleation process, its maximal rate is reached when both species are populated; thus secondary nucleation is most active in the vicinity of the half time of the aggregation reaction (Fig. 11), in the region which is typically defined as “growth phase”. This observation highlights the fact that the lag phase should not be associated univocally to a nucleation period, since in the presence of secondary nucleation processes nucleation events are more frequent in the growth phase.

Taken together, these considerations show that nucleation occurs from very early in the aggregation reaction and that the duration of the lag time is in most cases not strongly influenced by the primary nucleation rate, as would be tempting to assume for a simple sequential process, but rather by the amplification of primary nuclei through their growth and proliferation by secondary nucleation processes. A corollary of this idea is that amyloid fibrils would be expected to be found in solution also during the lag phase, albeit at very low concentrations. Conventional bulk assays are not sufficiently sensitive to detect such low concentrations, but fibrils in the nanomolar concentration range can indeed be detected at least a few minutes into the lag phase using amyloid chain amplification methods.^32^

### 7.3 Sensitivity of the lag-phase to different microscopic processes

**Fig. 12:**
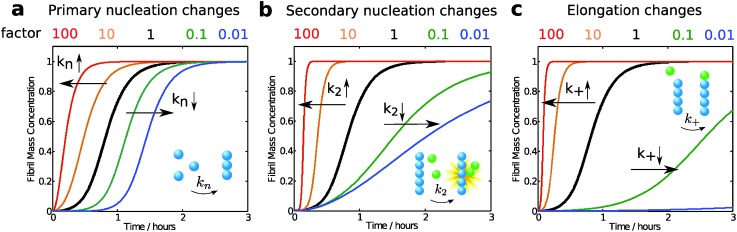
Effect of individual rate constants on macroscopic aggregation growth curves. In each panel the black curve represents the simulated time evolution of the fibril mass *versus* time for a 4 μM solution of Aβ42 in 20 mM phosphate buffer, 0.2 mM EDTA, 0.02% NaN_3_ at pH 8.0 under quiescent conditions according to the following rate constants: *k*_*n*_ *k*_+_ = 900 M^–2^ s^–2^; *k*_2_ *k*_+_ = 4 × 10^10^ M^–3^ s^–2^, and nucleus size *n*_C_ = *n*_2_ = 2. (a) The original calculated curve (black) and curves generated by increasing (*k*_*n*_ *k*_+_ = 9 × 10^3^ M^–2^ s^–2^; orange and *k*_*n*_ *k*_+_ = 9 × 10^4^ M^–2^ s^–2^; red) or decreasing (*k*_*n*_ *k*_+_ = 90 M^–2^ s^–2^, green; *k*_*n*_ *k*_+_ = 9 M^–2^ s^–2^, blue) the rate constant for primary nucleation by a factor of 10 or 100. (b) The original curve (black) and curves generated by increasing (*k*_2_ *k*_+_ = 4 × 10^11^ M^–3^ s^–2^ orange; *k*_2_ *k*_+_ = 4 × 10^12^ M^–3^ s^–2^ red) or decreasing (*k*_2_ *k*_+_ = 4 × 10^9^ M^–3^ s^–2^ green; *k*_2_ *k*_+_ = 4 × 10^8^ M^–3^ s^–2^ blue) the rate constant for secondary nucleation by a factor of 10 or 100. (c) The original curve (black) and curves generated by increasing (*k*_*n*_ *k*_+_ = 9 × 10^3^ M^–2^ s^–2^ and *k*_2_ *k*_+_ = 4 × 10^11^ M^–3^ s^–2^ orange; *k*_*n*_ *k*_+_ = 9 × 10^4^ M^–2^ s^–2^ and *k*_2_ *k*_+_ = 4 × 10^12^ M^–3^ s^–2^ red) or decreasing (*k*_*n*_ *k*_+_ = 90 M^–2^ s^–2^, *k*_2_ *k*_+_ = 4 × 10^9^ M^–3^ s^–2^, green; *k*_*n*_ *k*_+_ = 9 M^–2^ s^–2^, 4 × 10^8^ M^–3^ s^–2^, *k*_2_ *k*_+_ = 4 × 10^8^ M^–3^ s^–2^, blue) the rate constant for elongation by a factor of 10 or 100.

Modifications of each microscopic process influence the overall growth curve in different characteristic manners and to different extents. To illustrate this concept, we have compared the aggregation curve for a 4 μM solution of Aβ42 using the rate constants obtained in 20 mM sodium phosphate at pH 8.0, with 0.2 mM EDTA, 0.02% NaN_3_^17^ with the curves generated by increasing or decreasing by a factor of 10 or 100 each single rate constant (Fig. 12). In this approach, the apparent rate constants considered in the simulations describe any modification of the reaction rate of the corresponding microscopic process, which may occur by alteration of either the intrinsic rate constant associated to the reaction or the concentrations of the species participating to the reaction (Fig. 9).

As seen in Fig. 12, the rate constant for primary nucleation has an influence on the length of the lag phase, which is reduced by half at 10 fold higher rate and is doubled at 10-fold reduced rate constant. This observation is intuitively easy to understand, since nucleation is absolutely required for aggregates to emerge. However, the aggregation curves shown in Fig. 12 highlight the fact that elongation and secondary nucleation rates may have equally strong or even a stronger influence on the length of the lag phase, which decreases or increases if these reactions are, respectively, increased or decreased. In particular, changes in the elongation rate constant have dramatically larger effects on the duration of the lag phase with respect to similar changes in the primary nucleation constant. These key observations confirm once more that the lag phase has to be associated to the amplification of primary nuclei through their growth and proliferation by elongation and secondary nucleation processes, and should not be considered as a nucleation time only. In addition we note that modifications of the primary nucleation rate do not affect the growth phase, while changes in elongation and secondary nucleation modify both the lag phase and the growth phase.

## 8. Factors affecting the length of the lag phase

A vast number of studies have reported molecular factors (intrinsic or extrinsic) that influence the different microscopic reactions and therefore the length of the lag phase. Several of these factors have been associated to the onset and progress of the diseases. Therefore, elucidating their role in the microscopic reaction pathway can provide insights into the connection between the aggregation process and the pathology. Here we limit to discuss the fundamental physico-chemical aspects underlying the effect of these factors on the lag-phase, while the connection between these factors and their role in the disorders is highly disease-specific and will not be addressed here. Intrinsic factors found to influence the length of the lag-phase include sequence variants such as mutations, truncation and extensions.^55–59^ Extrinsic factors include peptides and proteins,^60–69^ membranes,^70,71^ nanoparticles and other surfaces,^72–81^ poly-electrolytes and other polymers,^82,83^ salt,^43,84–86^ small molecules,^87,88^ pH,^54,70^ temperature or mechanical factors such as shear imposed by for example shaking.^17,89,90^ Foreign surfaces, *e.g.* presented on nanoparticles, can either catalyse or inhibit aggregation, leading to shortening or lengthening of the lag phase^72–75,77^ depending on the protein to surface area ratio and whether the surface is weakly or strongly attractive.^79^

Based on the previous discussion, the different molecular factors listed above can influence the duration of the lag phase by modifying different microscopic events. Therefore, changes in the lag phase should not be associated directly to modifications of the primary nucleation rate, since elongation and secondary nucleation rate have a dramatic effect on the duration of the lag-phase, as illustrated in Fig. 12. For instance, the length of the lag phase is dramatically decreased if the fragmentation rate increases, which may happen if samples are shaken,^17^ or if the mechanical stability of the fibrils is reduced due to for example impeded inter-peptide interactions within the fibril. This is the reason why mechanical agitation is a popular method to reduce the length of the lag phase and bring the reaction into a more readily accessible time frame.

For the different molecular factors, only a limited set of studies have assigned the observed macroscopic effects on the length of the lag phase to modifications of specific microscopic processes.^91,92^ In this context, kinetic analysis is emerging as a powerful tool to correlate changes in the macroscopic reaction profiles to modifications of the molecular events.^93^

We note that different physicochemical effects can underlie the molecular mechanisms responsible for the modification of the microscopic reactions by the different molecular factors. For instance, reaction rate constants depend on intrinsic properties of the aggregating protein, such as the net charge (the more charged the more self-repulsion) and the surface hydrophobicity, as well as on the solution composition in terms of pH (modulation of charges), salt (screening of self-repulsion) and the presence of other proteins, membranes and other surfaces, which that may serve to enhance or interfere with the different microscopic processes. As a consequence, the different molecular factors can modify the microscopic reaction rates by affecting directly the intrinsic reaction rate constant associated to a specific microscopic event.

In other cases, the molecular factors modify the reaction rate by changing the concentration of the reactive species rather than the intrinsic reaction rate constant. Indeed, compounds or proteins that bind monomers and lower the concentration of free monomers in solution lead to an extension of the lag phase in a concentration-dependent manner up to *ca.* 1 : 1 molar ratio depending on the affinity for monomers. One striking example in this category is the affibody Z_a_β_3_ selected from a phage display library against Aβ-monomers.^62^ Other factors inhibit the aggregation process by interacting with aggregates rather than with monomers. Examples include proteins that extend the lag phase at low sub-stoichiometric levels by interacting with growing aggregates^67,91,94^ as well as other factors, such as the molecular chaperone Brichos, which bind selectively to the surface of the fibrils, thereby inhibiting secondary nucleation events. This latter case is particularly intriguing, since this modulation of the reaction mechanism not only leads to an extension of the lag phase (*t*_0.5_ increases by 100%, Fig. 12b) but, most importantly, redirects the reactive flux to a pathway that includes only primary nucleation and elongation, thereby resulting in longer fibrils and in a minimal generation of oligomers and toxicity.^92^

## 9. Outstanding questions

As discussed in the previous paragraphs, significant knowledge has emerged over the last years regarding the molecular events occurring during the lag phase of an amyloid formation process. However, there are still several outstanding questions open for the future, as illustrated by the following examples.

(i) A crucial issue refers to the characterization of the oligomers accumulating during the lag-phase. This question is of particular relevance because these low molecular weight species are currently thought to exert toxic functions. As discussed above, according to one of the several possible definitions, oligomers are small aggregates exhibiting different structure and lower growth rate with respect to fibrils. Therefore, oligomers can accumulate during on-going reactions, in particular in the presence of secondary nucleation events, although they represent always a very small fraction of the species present in the system. The experimental methods discussed in paragraph 5 have been successfully applied for the quantification of monomers and fibrils. In contrast, there is a severe lack of experimental techniques able to quantify oligomers, largely because of the low concentration and the transient nature of these species.

Ideally, building on recent advances for *in situ* identification or *ex situ* quantification of oligomers,^17,92,95,96^ the development of new experimental approaches for the *in situ* quantification of these species will open the possibility to measure the reaction orders of the microscopic events leading to their formation. In analogy with the kinetic analysis performed with mature fibrils, the measurement of the reaction orders of reactions involving oligomers will provide crucial information on the microscopic reaction mechanisms leading to the generation of these species. Such kinetic analysis can potentially explain why oligomers accumulate during the aggregation process, and which types of structural conversions they undergo. These answers can clarify for instance if the globular aggregates (“globulomers”) observed by TEM during the lag phase for several different synthetic peptides are a result of structural incompatibility and frustration in these sequence-inhomogeneous systems, or whether such globulomers could be also found in pure samples of recombinant or biologically derived peptides with high sequence homogeneity. Overall, improving our mechanistic understanding of the formation of oligomers could provide insights into the detailed molecular connection between the lag phase (and more in general the entire aggregation process) with the pathogenesis of most amyloid diseases.

(ii) Another outstanding question regards the molecular events occurring during the lag phase of aggregation reactions in complex mixtures. Indeed, bottom-up studies use clean systems that are challenged by single chemical or physical perturbations. Conversely, top-down studies jump all the way to aggregation reactions in biological fluids with their huge complexity. From a combination of these two approaches, it may be possible to address the molecular events occurring during the lag phase in a complex environment, and deconvolute the observed effects into the underlying perturbations resulting from long-range interactions (such as, for example, electrostatic screening) and from short-range interactions, including for instance the binding of molecules to various species along the reaction pathway.

(iii) Lateral aggregation of proto-fibrils and fibrils is observed in a large variety of amyloid systems. It would be important to clarify how this additional microscopic process affects elongation and nucleation processes, and the corresponding consequences on the duration of the lag-phase.

(iv) Lastly, since secondary nucleation of monomers on the fibril surface dominates over primary nucleation during most of the lag phase in different amyloid systems, including Aβ42, it would be highly interesting to study the generality of this microscopic process in amyloid formation reactions of various proteins and peptides, including not only pathological but also functional amyloids.

## 10. Conclusions

In summary, we have discussed the nature and the molecular origin of the macroscopic lag-phase that is typically observed during the formation of amyloid fibrils from a solution of soluble monomeric peptides or proteins. After presenting currently available physicochemical experimental techniques to measure the lag-phase, we have discussed how the lag-phase should not be interpreted as a waiting time for nuclei to form. Indeed, this type of collective waiting behavior is possible only for single-molecule reactions, while typically millions of primary nuclei form during the lag-phase in macroscopic samples. Rather, the lag-phase results from the combination of multiple parallel microscopic reactions, and must be associated to the amplification of primary nuclei through their growth and proliferation by secondary nucleation and fragmentation processes.
